# α,β-Aziridinylphosphonates by lithium amide-induced phosphonyl migration from nitrogen to carbon in terminal aziridines

**DOI:** 10.3762/bjoc.6.110

**Published:** 2010-10-13

**Authors:** David M Hodgson, Zhaoqing Xu

**Affiliations:** 1 Department of Chemistry, Chemistry Research Laboratory, University of Oxford, Mansfield Road, Oxford, OX1 3TA, UK, Fax: +44(1865) 285002

**Keywords:** amino acids, aziridines, lithiation, migration, synthetic methods

## Abstract

*N*-Phosphonate terminal aziridines undergo lithium 2,2,6,6-tetramethylpiperidide-induced *N-* to *C-*[1,2]-anionic phosphonyl group migration under experimentally straightforward conditions, to provide a stereocontrolled access to synthetically valuable *trans*-α,β-aziridinylphosphonates. The utility of this chemistry has been demonstrated in the asymmetric synthesis of a β-aminophosphonate.

## Introduction

**Scheme 1:**
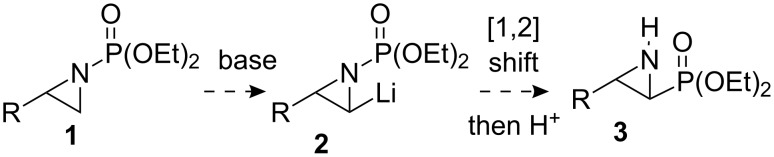
Proposed aziridinyl anion induced *N*- to *C*-phosphonyl migration.

The synthesis of aminophosphonic acids and their derivatives has attracted considerable attention, since the presence of such functionality, typically as amino acid surrogates, leads to interesting bioactivity in, for example, antibacterial agents, enzyme inhibitors and herbicides [1–3]. α,β-Aziridinylphosphonates **3**, while possessing biological activity themselves, can be converted into aminophosphonic acids using various ring-opening processes [4–6]. Aziridinylphosphonates **3** have previously been prepared by a variety of methods [4–6], of which ring-closure following either Sharpless aminohydroxylation of α,β-unsaturated phosphonates [7], or α-halophosphonate addition to sulfinimines [8] constitute notable asymmetric approaches, albeit principally leading to β-aryl substituted α,β-aziridinylphosphonates. Arising from our investigations [9–13] on the generation and subsequent chemistry of α-lithiated terminal aziridines [14], we considered whether α,β-aziridinylphosphonates **3** could be accessed by α-lithiation of *N*-phosphonate terminal aziridines **1**, followed by *N*- to *C*-[1,2]-anionic phosphonyl group migration in lithiated intermediate **2** (Scheme 1). Here, we present full details of this study [15].

**Scheme 2:**
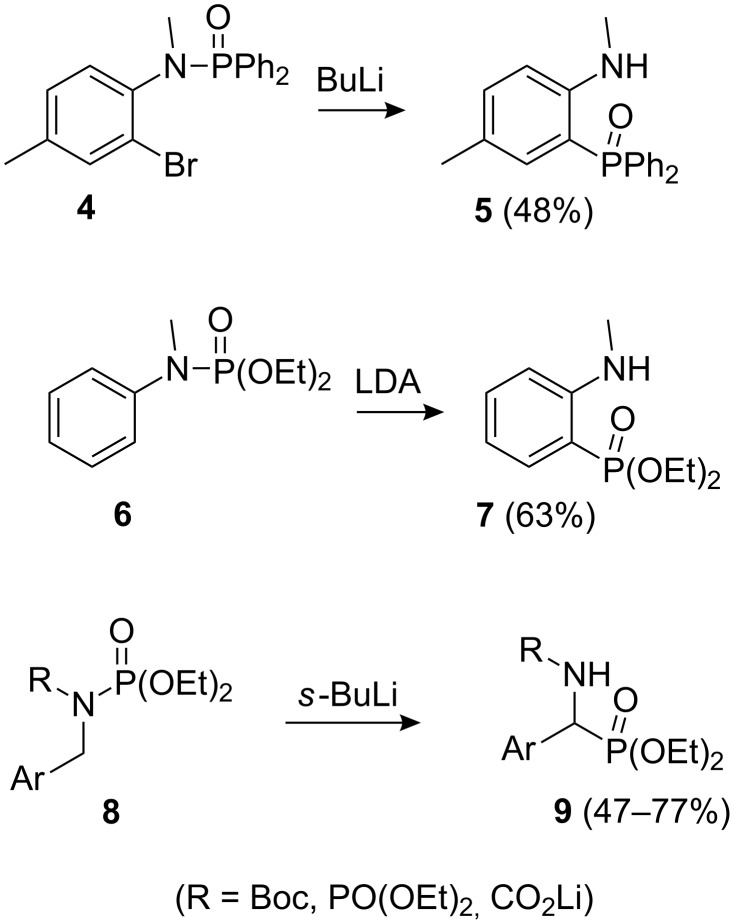
Selected previously observed *N*- to *C*-phosphorous migrations [17–18,21].

Based-induced migration, involving cleavage of a nitrogen phosphorus bond and formation of a carbon phosphorus bond, was first reported over 30 years ago by Hellwinkel and co-workers (**4**→**5**, Scheme 2) [16–17]. More recently, Modro et al. reported a similar *ortho*-lithiation followed by [1,3]-shift of a phosphonyl group (**6**→**7**, Scheme 2) [18]; related processes have been observed with diazaphospholidine oxides [19] and bicyclic phosphoric triamides [20]. Benzylic lithiation-induced *N*- to *C*-[1,2]-anionic phosphonyl rearrangements (**8**→**9**, Scheme 2) were developed by Hammerschmidt and Hanbauer [21], and a stereoretentive *N*- to *C*-[1,2]-shift in an α-lithiated pyrrole involving a chiral *tert*-butyl(phenyl)phosphinyl group has been reported [22].

**Scheme 3:**
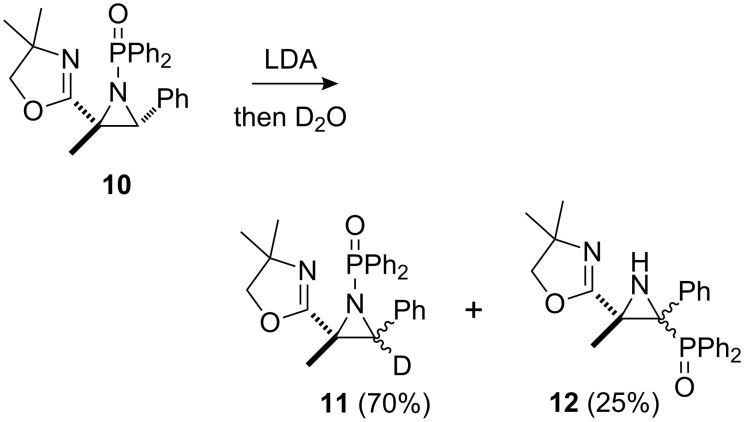
Partial *N*- to *C*-migration with *N*-diphenylphosphinylaziridine **10** [24].

With regard to previous anion-induced *N*- to *C*-1,2-shifts in aziridines, in one isolated example, the *N*-Boc-aziridine of styrene was treated with *s*-BuLi in THF at –98 °C to give a phenyl-stabilised α-lithiated aziridine, which underwent migration to give 2-phenyl-2-Boc-aziridine (90%) [23]. Also, our laboratory has reported the LTMP (lithium 2,2,6,6-tetramethylpiperidide)-induced rearrangement of a range of terminal *N*-Boc-aziridines to give *trans*-aziridinyl esters [12,15] (cf. Scheme 1, with CO_2_*t*-Bu instead of PO(OEt)_2_). However, prior to our studies only one example of a *N-* to *C*-[1,2]-anionic rearrangement of an aziridine involving phosphorus had been observed: lithiation-deuteration of *N*-diphenylphosphinylaziridine **10** gave the anticipated deuterated aziridine **11** (70%), along with the rearranged aziridine **12** (25%) [24] (Scheme 3).

## Results and Discussion

**Scheme 4:**
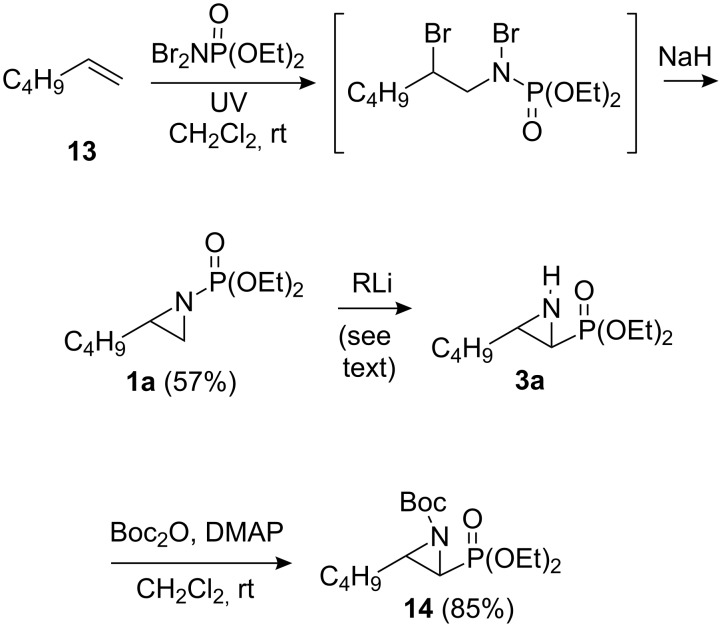
Synthesis and rearrangement of aziridine **1a**.

So as to examine the migration chemistry outlined in Scheme 1, access to *N*-phosphonate terminal aziridines **1** was required [25]. These can be concisely prepared from alkenes using chemistry developed by Zwierzak and co-workers [26–28]. In a one-flask operation, addition of 1-hexene (**13**) to Br_2_NPO(OEt)_2_ [29] in the presence of UV light [27] and subsequent treatment with NaH (2 equiv) [26] gave the representative substrate **1a** (57%, Scheme 4) [28].

Initially, we examined organolithiums for their propensity to induce deprotonation-migration in *N*-phosphonate aziridine **1a**. Despite it being previously noted by Zwierzak that the reaction of organolithium reagents with such substrates resulted in preferential attack at phosphorus giving a complex mixture of products [30], we did obtain some of the desired rearrangement product **3a** (Scheme 4). When aziridine **1a** in THF was treated with *n*-BuLi (1.5 equiv) at −78 °C for 4 h, 46% of rearrangement product **3a** was obtained (66% based on recovered **1a**). The stereochemistry of aziridinylphosphonate **3a** was assigned as *trans* on the basis of the small sizes of the vicinal H-H couplings across the ring in **3a** and in the derived *N*-Boc aziridinylphosphonate **14** (3–3.5 Hz), which are diagnostic for such systems (the corresponding *cis*-aziridinylphosphonates are known to have larger ^3^*J* values of 6–7 Hz) [31–32]. Generation of *trans*-stereochemistry was also observed in the corresponding *N*-Boc system [12,15] (see earlier discussion), and the present transformation likely follows a similar reaction pathway: initial trans α-lithiation, which is probably assisted by prior complexation of the base with the *N*-protecting group, followed by intramolecular migration [12] of this group.

Reactions of *s*-BuLi or *t*-BuLi with aziridine **1a** under the same conditions as above were less effective, giving a 22% yield of **3a** (50% based on recovered **1a**) and only traces of **3a** (45% **1a** recovered), respectively. In an attempt to induce greater conversion of **1a** with *n*-BuLi, the amount of the latter was increased to 3 equivalents, however only 25% of **3a** was obtained. Given previous successes with the use of lithium amides to deprotonate differently *N*-protected terminal aziridines [9–14], we moved on to study such bases with *N*-phosphonate aziridine **1a**. Firstly, LDA (lithium diisopropylamid) and LiNCy_2_ were examined. Although there was no significant increase in yield on using LDA or LiNCy_2_, (38% and 15% of **3a**, respectively), under the same conditions as for *n*-BuLi described above, side-reactions were not observed. These results prompted us to try the stronger lithium amide, LTMP. Initial studies indicated that with 3 equiv of LTMP, **3a** was obtained in 52% yield (94% based on recovered **1a**). Prolonging the reaction time from 4 to 8 h made no difference to the yield. However, when the amount of LTMP was increased to 4 equiv and to 5 equiv, **3a** was obtained in 65% and 91% yields, respectively. These latter conditions were then applied to a range of terminal *N*-phosphonate aziridines **1** (Table 1).

**Table 1:** Aziridinylphosphonate **3** synthesis.

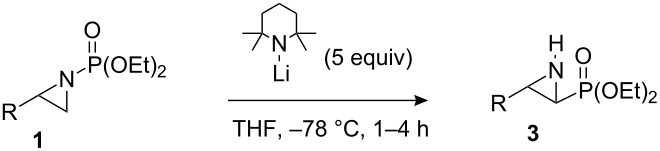			

Entry	Aziridine **1**	Aziridinylphosphonate **3**	Yield (%)

1	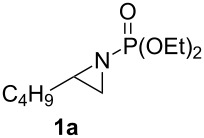	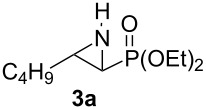	91
2	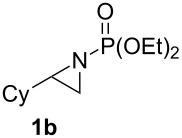	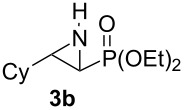	79
3	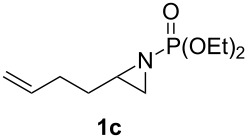	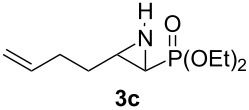	79
4	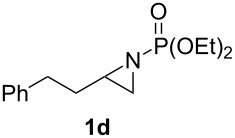	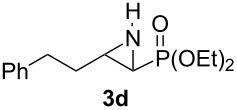	87
5	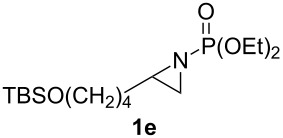	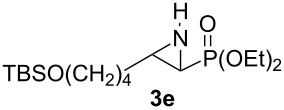	95
6	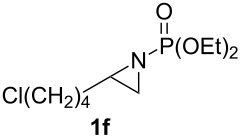	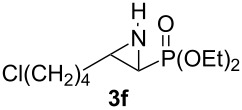	87
7	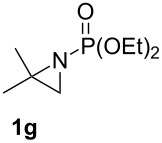	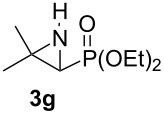	58

**Figure 1:**
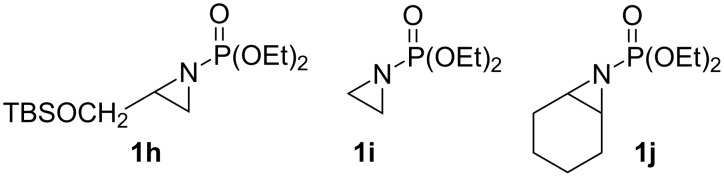
Aziridines **1h**–**j**.

Terminal aziridine **1b**, possessing secondary alkyl substitution on the aziridine, gave the rearranged aziridinylphosphonate **3b** in 79% yield (Table 1, entry 2). Aziridines **1c** and **1d** underwent migration smoothly without complications arising from potential allylic deprotonation [33], intramolecular cyclopropanation [11–12] or benzylic deprotonation (entries 3 and 4). Mixed results were obtained when the method was applied to substrates possessing silyl ether functionality. The distal-protected aziridine **1e** provided stable aziridinylphosphonate **3e** in high yield (entry 5), whereas rapid decomposition was observed during attempted purification of the product mixture from the proximal-protected substrate **1h** (Figure 1). However, the migration reaction could be used to prepare aziridinylphosphonate **3f** (entry 6), without potential elimination [34] of the primary chloride. From 2-chloroethylamine hydrochloride, the simplest *N*-phosphonate aziridine **1i** (Figure 1) could be straightforwardly accessed [35]. However, this latter substrate decomposed under the lithiation conditions. A 2,2-disubstituted *N*-phosphonate aziridine **1g**, available from isobutene and Br_2_NPO(OEt)_2_ without UV assistance followed by methanolic NaOMe-induced ring-closure [26–27,30], proved viable in the lithiation [1,2]-shift chemistry giving aziridinylphosphonate **3g** (58%, Table 1, entry 7). However, and similarly to the *N*-Boc aziridine of cyclohexene [12,15], no reaction was observed between LTMP and a 2,3-disubstituted aziridine **1j** (Figure 1), presumably due to steric interactions and/or reduced acidity impeding lithiation.

LDA has been reported to rapidly (THF, –78 °C, 10 min) isomerize *N*-Ph *cis*-β-phenyl-aziridinylphosphonate to mainly (80:20) the *trans*-isomer **3** (NPh instead of NH, and R = Ph) via an α-deprotonation pathway [36]. Therefore, although substrate and/or product co-ordination and/or LTMP aggregation phenomena could be invoked to rationalise the beneficial effect of the excess LTMP on yield, and we viewed α-deprotonation as more difficult in our likely post-rearrangement intermediate **3** (N–Li instead of N–H, Scheme 1), we sought to rule out the possibility that LTMP might be being consumed by further lithiation in the rearranged product. In the event, quenching a migration reaction of *N*-phosphonate aziridine **1a** with CD_3_OD did not show any deuterium incorporation. This prompted a closer investigation of the reaction using a more rigorously purified sample of aziridine **1a** than which had been used in our preliminary studies. This latter work established that direct application of our earlier *N*-Boc migration conditions (3 equiv LTMP, –78 °C, 90 min) [12] gave complete consumption of the starting aziridine **1a**, with aziridinylphosphonate **3a** being isolated in 82% yield (use of 2 equiv LTMP gave **3a** in 68% yield, with 7% recovered **1a**) and suggests that 5 equiv of the base may not always be needed to effect efficient rearrangement.

**Scheme 5:**
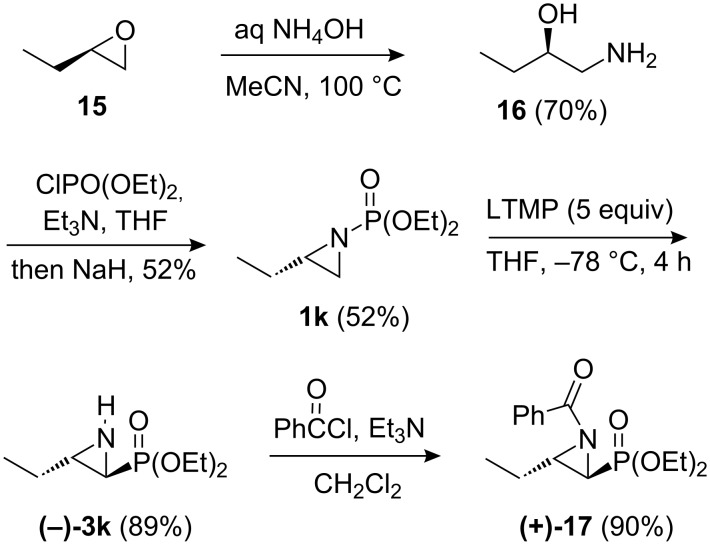
Synthesis and rearrangement of aziridine (*S*)-**1k**.

Asymmetric access to an aziridinylphosphonate was also explored to demonstrate further the utility of the above methodology. As terminal epoxides are readily available as single enantiomers [37], terminal epoxide-opening with H_2_NPO(OEt)_2_ followed by ring-closure was initially considered as a potential asymmetric route to *N*-phosphonate terminal aziridines **1**. However, 1,2-epoxyhexane could not be successfully ring-opened with H_2_NPO(OEt)_2_ under a variety of conditions (NaH, KH, or NaH/DMPU in THF; (*i*-PrO)_4_Ti or BF_3_•Et_2_O in CH_2_Cl_2_; aminolytic kinetic resolution [12,38]), and use of (Me_3_)SiHNPO(OEt)_2_ [39] also proved ineffective. A convenient asymmetric access was eventually developed, starting with ring-opening of commercially available (*R*)-1,2-epoxybutane (**15**) using ammonia, which gave β-amino alcohol **16** in good yield (Scheme 5) [40]. By analogy with a preparation of *N*-diphenylphosphinyl aziridines [41], subsequent one-pot *N*- and *O*-phosphonylation followed by NaH-induced ring-closure gave (*S*)-aziridine **1k** (52%). Lithiation–rearrangement of (*S*)-aziridine **1k** provided aziridinylphosphonate (–)-**3k** in excellent yield (89%), and without any degradation of enantiopurity (>99% ee, as determined by chiral HPLC analysis of the benzoyl derivative **17**).

**Scheme 6:**
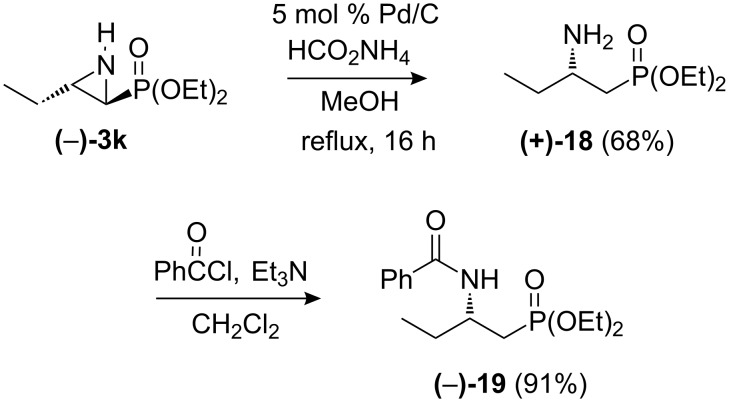
Hydrogenolysis of aziridinylphosphonate (–)-**3k**.

Hydrogenolytic ring-opening of aziridinylphosphonates provides an attractive entry to α- or β-aminophosphonates [6]. α,β-Aziridinylphosphonates bearing a β-aryl group undergo cleavage at the formally benzylic C–N bond, leading to α-aminophosphonates. With β-alkyl groups, regioselectivity is influenced by the presence or absence of an *N*-substituent. *N*-Ts *Cis*-β-alkyl-substituted aziridinylphosphonates give β-aminophosphonates [32], whereas both *N*-Boc *cis*- and *trans*-β-alkyl-substituted aziridinylphosphonates lead to α-aminophosphonates. For example, hydrogenolysis of *N*-Boc *trans*-aziridinylphosphonate **14** (Bn instead of the C_4_H_9_ group) using 10% Pd/C (H_2_ (1 atm), EtOH, 12 h) has been reported to give the corresponding *N*-Boc α-aminophosphonate (63%) [31]. In the absence of an *N*-substituent, *cis*-β-alkyl-substituted aziridinylphosphonates give β-aminophosphonates [32] and we observed that *trans*-β-alkyl-substituted aziridinylphosphonate (–)-**3k** underwent completely regioselective hydrogenolysis under transfer hydrogenation conditions [31] to produce β-aminophosphonate (+)-**18** [32,42] in 68% yield with >99% ee (determined by chiral HPLC analysis of the benzoyl derivative **19**, Scheme 6).

## Conclusion

In summary, lithiation-induced phosphonyl migration from nitrogen to carbon in terminal aziridines **1** effects simultaneous *N*-deprotection and accesses synthetically valuble N–H *trans*-aziridinylphosphonates **3**. The process is experimentally straightforward to carry out and occurs in an efficient and completely stereocontrolled fashion. The utility of the chemistry is further highlighted by a subsequent conversion into an enantiopure β-aminophosphonate **18**, demonstrating an entry to a biologically important β-amino acid mimic class.

## Supporting Information

Full preparative details of all compounds are reported, together with their spectroscopic data.

File 1 Experimental and analytical data
