# Supplementary material for: α,β-Aziridinylphosphonates by lithium amide-induced phosphonyl migration from nitrogen to carbon in terminal aziridines
Source: Beilstein J Org Chem. 2010 Oct 13;6:978–83. doi: 10.3762/bjoc.6.110 (PMC2981828; doi:10.3762/bjoc.6.110)
Supplement: File 1 — Experimental and analytical data [file Beilstein_J_Org_Chem-06-978-s001.pdf]

Supporting Information

for

α,β-Aziridinylphosphonates by lithium amide-induced phosphonyl migration from nitrogen to carbon in terminal aziridines

David M. Hodgson* and Zhaoqing Xu

Department of Chemistry, Chemistry Research Laboratory, University of Oxford, Mansfield Road, Oxford, OX1 3TA, UK; Fax +44(1865) 285002

Email: David M. Hodgson* - david.hodgson@chem.ox.ac.uk

* Corresponding author

Experimental and analytical data

Table of contents

Page

S3 General remarks

S4 General procedure A: synthesis of *N*-phosphonate aziridines **1**

S4 Diethyl 2-butylaziridin-1-ylphosphonate **1a**

S5 Diethyl 2-cyclohexylaziridin-1-ylphosphonate **1b**

S5 Diethyl 2-(but-3-enyl)aziridin-1-ylphosphonate **1c**

S6 Diethyl 2-phenethylaziridin-1-ylphosphonate **1d**

S6 Diethyl 2-[4-(*tert*-butyldimethylsilyloxy)butyl]aziridin-1-ylphosphonate **1e**

S7 Diethyl 2-(4-chlorobutyl)aziridin-1-ylphosphonate **1f**

S7 Diethyl 2,2-dimethylaziridin-1-ylphosphonate **1g**

S8 Diethyl 2-[(*tert*-butyldimethylsilyloxy)methyl]aziridin-1-ylphosphonate **1h**

S9 Diethyl aziridin-1-ylphosphonate **1i**

S9 Diethyl 7-azabicyclo[4.1.0]heptan-7-ylphosphonate **1j**

S10 General procedure B: Synthesis of *N*-H aziridinylphosphonates **3**

S10 Diethyl (2*R**,3*R**)-3-butylaziridin-2-ylphosphonate **3a**

S11 *tert*-Butyl (2*R**,3*R**)-2-butyl-3-(diethoxyphosphoryl) aziridine-1-carboxylate **14**

S11 Diethyl (2*R**,3*R**)-3-(cyclohexyl)aziridin-2-ylphosphonate **3b**

S12 Diethyl (2*R**,3*R**)-but-3-enylaziridin-2-ylphosphonate **3c**

S12 Diethyl (2*R**,3*R**)-3-(phenethyl)aziridin-2-ylphosphonate **3d**

S13 Diethyl (2*R**,3*R**)-3-[4-(*tert*-butyldimethylsilyloxy)butyl]-aziridin-2-ylphosphonate **3e**

S14 Diethyl (2*R**,3*R**)-3-(4-chlorobutyl)aziridin-2-ylphosphonate **3f**

S14 Diethyl 3,3-dimethylaziridin-2-ylphosphonate **3g**

S15 (*R*)-1-Aminobutan-2-ol **16**

S15 Diethyl (*S*)-2-ethylaziridin-1-ylphosphonate **1k**

S16 Diethyl (2*S*,3*S*)-3-ethylaziridin-2-ylphosphonate **(–)-3k**

S17 Diethyl (2*S*,3*S*)-1-benzoyl-3-ethylaziridin-2-ylphosphonate **(+)-17**

S18 Diethyl (*S*)-(2-aminobutyl)phosphonate **(+)-18**

S19 Diethyl (*S*)-2-benzamidobutylphosphonate **(–)-19**

S20 References

General remarks

All reactions requiring anhydrous conditions were performed in oven-dried or flame-dried glassware under an inert atmosphere (argon or nitrogen). CH_2Cl_2, Et_2O, and THF were degassed and dried over alumina, according to the procedure of Grubbs and co-workers [1]. 2,2,6,6-Tetramethylpiperidine was distilled from CaH_2 under reduced pressure. All other reagents were used as received unless otherwise stated. Reactions were monitored by TLC, using Merck aluminium-backed plates pre-coated with silica (0.25 mm, 60, F_{254}). The plates were visualised under UV light and developed using a solution of basic $KMnO_4$. Removal of solvent under reduced pressure was performed using Büchi rotary evaporators, achieving a minimum pressure of ca. 15 mbar, followed by drying at 0.1 mbar using an oil pump. Column chromatography was performed on silica [Kieselgel 60 (40–63 μm)]. Petroleum ether refers to the fraction boiling in the range 30–40 °C. Optical rotations were measured using a Perkin-Elmer 241 polarimeter with a cell of path length 10.0 cm; concentrations are quoted in g/100 mL; specific rotations are given in 10^{-1} deg cm^2 g^{-1}. Infrared spectra were recorded on a Perkin-Elmer 1750 FTIR or a Bruker Tenso 27 FTIR spectrometer; absorptions are quoted in wave numbers (cm^{-1}) and are classified as s (strong), m (medium), w (weak) and/or br (broad); only selected absorptions are recorded. ^1H,^{13}C and ^{31}P NMR spectra were recorded on Bruker DPX 200, DPX 250, DPX 400, DQX 400 or AMX 500 spectrometers; chemical shifts (δ) are quoted in parts per million, referenced to the residual solvent peak as an internal standard [2]; coupling constants (*J*) are quoted in Hz. High-resolution mass spectra were obtained by chemical ionisation (NH_3 and Na^+) or by GC analysis with a BPX5 column-HP 6890 (dimethyl silicone capillary column, l = 30 m, ϕ = 0.25 mm) equipped with a reflectron TOF mass spectrometer (60 eV, He flow rate = 1 mL min^{-1}). Chiral GC analyses were carried out using a CE Instruments Trace GC (Thermoquest) chromatograph, fitted with an SGE Cydex-B column. Chiral HPLC analyses were carried out using Daicel Chiracel OD, AD or OJ columns (l = 250 mm, diameter = 4.6 mm) on a Gilson System with 712 controller software and 188 UV/vis detector, operating at 255 or 224 nm.

General procedure A: synthesis of *N*-phosphonate aziridines 1 [3]

Alkene (3 mmol) was added to a stirred solution of $Br_2NPO(OEt)_2$ [4] (3 mmol) in CH_2Cl_2 (15 mL) under argon at room temperature. Following stirring for 4 h under UV irradiation (256 nm) at room temperature, the reaction mixture was cooled to 0 °C and NaH (6 mmol) was added slowly. Following stirring at 0 °C for 30 min, the suspension was warmed to room temperature and stirred for a further 1 h. CH_2Cl_2 (10 mL) and H_2O (10 mL) were added and the organic phase was washed with H_2O (2 × 10 mL). The organic phase was dried ($MgSO_4$) and evaporated under reduced pressure. Purification of the residue by column chromatography (EtOAc/$CHCl_3$) gave the *N*-phosphonate aziridines **1**.

Diethyl 2-butylaziridin-1-ylphosphonate 1a

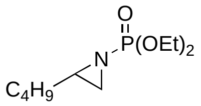

Following **General procedure A** using 1-hexene (0.64 g, 7.6 mmol) gave, following purification of the resulting residue by column chromatography (EtOAc/$CHCl_3$ 1:2), aziridine **1a** [5] as a pale yellow oil (1.02 g, 57%). R_f 0.54 (EtOAc/$CHCl_3$ 1:2); ν_{max}/cm^{-1} 2933s, 2873m, 1651w, 1467w, 1394w, 1264s (P=O), 1034s and 967s; ^1H NMR (400 MHz, $CDCl_3$) δ 4.17–4.10 (4H, m, 2 × OCH_2), 2.35–2.33 (1H, m, NCH), 2.31 (1H, dd, *J* 18, 6, CH(*H*)N), 1.88 (1H, dd, *J* 10, 4, C*H*(H)N), 1.51–1.36 (6H, m, 3 × CH_2), 1.33 (6H, t, *J* 7, CH_3) and 0.90 (3H, t, *J* 7, CH_3); ^{13}C NMR (100 MHz, $CDCl_3$) δ 63.2 (2 × $POCH_2$, t, $J_{C\text{-}P}$ 8), 36.8 (CHN, d, $J_{C\text{-}P}$ 7), 32.2 (*C*H_2CH), 30.8 (NCH_2, d, $J_{C\text{-}P}$ 7), 29.0 (CH_2), 22.3 (*C*H_2CH_3), 16.3 (2 × OCH_2*C*H_3, t, $J_{C\text{-}P}$ 6) and 13.9 (CH_3); ^{31}P NMR (162 MHz, $CDCl_3$) δ 16.3; MS CI *m/z* 236 (M + H^+, 100), 220 (20), 193 (90), 166 (45), 150 (80), 136 (20) and 98 (35); HRMS calcd for $C_{10}H_{23}NO_3{}^{31}P$, 236.1416, found 236.1419.

Diethyl 2-cyclohexylaziridin-1-ylphosphonate 1b

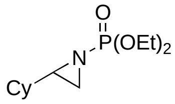

Following **General procedure A** using vinylcyclohexane (330 mg, 3.0 mmol) gave, following purification of the resulting residue by column chromatography (EtOAc/$CHCl_3$ 1:2), aziridine **1b** as a pale yellow oil (256 mg, 33%). R_f 0.6 (EtOAc/$CHCl_3$ 1:2); ν_{max}/cm^{-1} 2927s, 1645w, 1450m, 1260s (P=O), 1165m (P–O–C), 1034s and 979s; ^{1}H NMR (400 MHz, $CDCl_3$) δ 4.18–4.10 (4H, m, 2 × OCH_2), 2.28 (2H, m, CHN, CH(*H*)N), 1.92 (1H, dt, *J* 14, 5, 3, C*H*(H)N), 1.84 (1H, d, *J* 11, CH), 1.77–1.65 (4H, m, 2 × CH_2), 1.33 (6H, t, *J* 7, 2 × OCH_3) and 1.23–1.08 (6H, m, 3 × CH_2); ^{13}C NMR (100 MHz, $CDCl_3$) δ 63.2 (2 × OCH_2, t, $J_{C\text{-}P}$ 7), 41.8 (CHN, d, $J_{C\text{-}P}$ 6), 40.5 (CH, d, $J_{C\text{-}P}$ 5), 30.6 (2 × CH_2), 29.7 (CH_2N, t, $J_{C\text{-}P}$ 7), 26.2 (2 × CH_2), 25.7(CH_2) and 16.3 (2 × CH_3, d, $J_{C\text{-}P}$ 6); ^{31}P NMR(162 MHz, $CDCl_3$) δ 15.40; MS CI *m/z* 262 ($M+H^+$, 100); HRMS calcd for $C_{12}H_{25}NO_3{}^{31}P$, 262.1572, found 262.1581.

Diethyl 2-(but-3-enyl)aziridin-1-ylphosphonate 1c

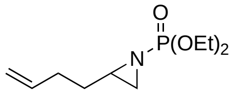

Following **General procedure A** using 1,5-hexadiene (984 mg, 12.0 mmol, 2 equiv) gave, following purification of the resulting residue by column chromatography (EtOAc/$CHCl_3$ 1:2), aziridine **1c** as a pale yellow oil (505 mg, 36%). R_f 0.50 (EtOAc/$CHCl_3$ 1:2); ν_{max}/cm^{-1} 2933m, 1641s (C=C), 1369m, 1265s (P=O), 1032m and 800m; ^{1}H NMR (400 MHz, $CDCl_3$) δ 5.87–5.77 (1H, m, =CH), 5.01 (2H, dd, *J* 17, 10, CH_2=), 4.17–4.10 (4H, m, 2 × OCH_2), 2.54–2.44 (1H, m, NCH), 2.32 (1H, dd, *J* 12, 6, CH(*H*)N), 2.23–2.16 (2H, m, =CHCH_2), 1.90 (1H, dd, *J* 11, 4, C*H*(H)N), 1.60–1.54 (2H, m, CH_2) and 1.33 (6H, t, *J* 7, 2 × CH_3); ^{13}C NMR (100 MHz, $CDCl_3$) δ 137.5 (=CH), 115.2 (CH_2=), 63.3 ($POCH_2$, d, $J_{C\text{-}P}$ 2), 63.2 ($POCH_2$, d, $J_{C\text{-}P}$ 2), 36.3 (CHN, d, $J_{C\text{-}P}$ 7), 31.8 (=CHCH_2, d, $J_{C\text{-}P}$ 6), 30.9 (NCH_2, t, $J_{C\text{-}P}$ 8 and CH_2) and 16.3 (2 × CH_2CH_3, d, $J_{C\text{-}P}$ 7); ^{31}P

NMR (162 MHz, $CDCl_3$) δ 15.2; MS CI *m/z* 234 ($M+H^+$, 95), 154 (30), 126 (15), 98 (100) and 94 (30); HRMS calcd for $C_{10}H_{21}NO_3{}^{31}P$, 234.1259, found 234.1251.

Diethyl 2-phenethylaziridin-1-ylphosphonate 1d

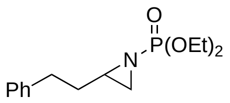

Following **General procedure A** using 4-phenyl–1-butene (933 mg, 3.0 mmol) gave, following purification of the resulting residue by column chromatography ($EtOAc/CHCl_3$ 1:2), aziridine **1d** as a pale yellow oil (600 mg, 71%). R_f 0.54 ($EtOAc/CHCl_3$ 1:2); ν_{max}/cm^{-1} 2985m, 1455m, 1394m, 1264s (P=O), 1163m (P–O–C), 1031s, 968s and 700m; ^1H NMR (400 MHz, $CDCl_3$) δ 7.31–7.20 (5H, m, 5 × $C_{Ar}H$) , 4.20–4.12 (4H, m, 2 × OCH_2), 2.85–2.70 (2H, m, $PhCH_2$), 2.57–2.48 (1H, m, NCH), 2.33 (1H, dd, *J* 18, 6, CH(*H*)N), 1.89 (1H, dd, *J* 14, 3, C*H*(H)N), 1.83–1.79 (2H, m, CH_2CH) and 1.35 (6H, t, *J* 7, 2 × CH_3); ^{13}C NMR (100 MHz, $CDCl_3$) δ 141.2 ($C_{Ar\ quat}$), 128.4 (2 × C_{Ar}), 128.3 (2 × C_{Ar}), 126.0 (C_{Ar}), 63.3 (2 × OCH_2, d, J_{C-P} 8), 36.4 (CHN, d, J_{C-P} 7), 34.4 (*C*H_2CHN), 33.2 (C_{Ar}*C*H_2), 30.9 (NCH_2, d, J_{C-P} 7) and 16.3 (2 × CH_3, d, J_{C-P} 6); ^{31}P NMR (162 MHz, $CDCl_3$) δ 14.69; MS CI *m/z* 284 (M + H^+, 100), 166 (5), 154 (20) and 148 (40); HRMS calcd for $C_{14}H_{23}NO_3{}^{31}P$, 284.1416, found 284.1408.

Diethyl 2-[4-(*tert*-butyldimethylsilyloxy)butyl]aziridin-1-ylphosphonate 1e

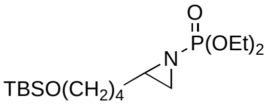

Following **General procedure A** using *tert*-butyl(hex-5-enyloxy)dimethylsilane [6] (642 mg, 3.0 mmol) gave, following purification of the resulting residue by column chromatography ($EtOAc/CHCl_3$ 1:2), aziridine **1e** as a pale yellow oil (193 mg, 18%). R_f 0.63 ($EtOAc/CHCl_3$ 1:2); ν_{max}/cm^{-1} 2931m, 2859s, 1472m, 1255s (P=O), 1103m (P–O–C) and 910s; ^1H NMR (400 MHz, $CDCl_3$) δ 4.18–4.11 (4H, m, 2 × OCH_2), 3.61 (2H, t, *J* 6, OCH_2), 2.51–2.44 (1H, m, NCH), 2.32

(1H, dd, J 12, 6, CH(H)N), 1.89 (1H, dd, J 10, 4, CH(H)N), 1.59–1.46 (6H, m, 3 × CH_2), 1.33 (6H, t, J 7, CH_3), 0.89 (9H, s, $C(CH_3)_3$) and 0.05 (6H, s, $Si(CH_3)_3$); ^{13}C NMR (100 MHz, $CDCl_3$) δ 63.2 (2 × $POCH_2$, t, $J_{C\text{-}P}$ 5), 62.9 (OCH_2), 36.8 (CHN, d, $J_{C\text{-}P}$ 6), 32.4 (2 × CH_2, t, $J_{C\text{-}P}$ 5), 30.9 (NCH_2, d, $J_{C\text{-}P}$ 7), 25.9 ($C(CH_3)_3$), 23.3 (CH_2), 18.3 (SiC), 16.3 (CH_2CH_3, d, $J_{C\text{-}P}$ 6) and -5.3 ($Si(CH_3)_2$); ^{31}P NMR (162 MHz, $CDCl_3$) δ 15.2; MS CI m/z 366 (M + H^+, 100), 351 (95), 234 (90), 228 (100) and 139 (40); HRMS calcd for $C_{16}H_{37}NO_4Si^{31}P$, 366.2230, found 366.2226.

Diethyl 2-(4-chlorobutyl)aziridin-1-ylphosphonate 1f

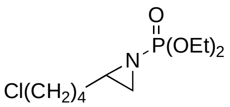

Following **General procedure A** using 6-chloro-1-hexene (356 mg, 3.0 mmol) gave, following purification of the resulting residue by column chromatography (EtOAc/$CHCl_3$ 1:2), aziridine **1f** as a pale yellow oil (555 mg, 69%). R_f 0.48 (EtOAc/$CHCl_3$ 1:2); ν_{max}/cm^{-1} 2986s, 1445m, 1394m, 1262s (P=O), 1164m, 1032s and 973s; ^{1}H NMR (400 MHz, $CDCl_3$) δ 4.18–4.11 (4H, m, 2 × OCH_2), 3.55 (2H, t, J 7, $ClCH_2$), 2.49–2.44 (1H, m, NCH), 2.33 (dd, J 10, 6, 1H CH(H)N), 1.90 (1H, dd, J 10, 4, CH(H)N), 1.86–1.80 (2H, m, $ClCH_2CH_2$), 1.63–1.40 (4H, m, 2 × CH_2) and 1.34 (6H, t, J 7, 2 × CH_3); ^{13}C NMR (100 MHz, $CDCl_3$) δ 63.3 (2 × OCH_2 d, $J_{C\text{-}P}$ 8), 44.8 ($ClCH_2$), 36.5 (CHN, d, $J_{C\text{-}P}$ 7), 32.0 (CH_2CH), 31.7 ($ClCH_2CH_2$, d, $J_{C\text{-}P}$ 5), 30.8 (NCH_2, d, $J_{C\text{-}P}$ 7), 24.3 (CH_2) and 16.3 (2 × CH_3 t, $J_{C\text{-}P}$ 6); ^{31}P NMR (162 MHz, $CDCl_3$) δ 16.1; MS CI m/z 272 (^{37}ClM + H^+, 15), 270 (^{35}Cl M + H^+, 100) and 234 (30); HRMS calcd for $C_{10}H_{22}NO_3{}^{31}P^{35}Cl$ 270.1026, found 270.1020.

Diethyl 2,2-dimethylaziridin-1-ylphosphonate 1g

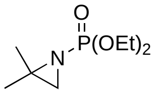

A solution of $Br_2NPO(OEt)_2$ [4] (1.87 g, 6.0 mmol) in CH_2Cl_2 (5 mL) was added dropwise to a saturated solution of isobutylene in CH_2Cl_2 (10mL) at room temperature at such a rate as to

maintain a slightly yellow colouration of the reaction mixture (~30 min). A slow stream of isobutylene was passed continuously through the solution and the resultant adduct was treated with NaOMe (15% solution in MeOH, 4.30 mL 12.0 mmol) at room temperature. Following stirring for 1 h, H_2O (10 mL) was added, the organic phase was washed with H_2O (2 × 10 mL), dried ($MgSO_4$) and evaporated under reduced pressure. Purification of the residue by column chromatography (EtOAc/$CHCl_3$ 1:2) gave aziridine **1g** [3,8] as a colourless oil (807 mg, 65%). R_f 0.55 (EtOAc/$CHCl_3$ 1:2); ν_{max}/cm^{-1} 2934m, 2360m, 1254s (P=O), 1149m (P–O–C) and 970s; ^{1}H NMR (400 MHz, $CDCl_3$) δ 4.15–4.08 (4H, m, 2 × OCH_2), 2.18 (1H, s, C(*H)*HN), 2.15 (1H, s, C(H)*H*N), 1.39 (6H, s, 2 × CH_3) and 1.32 (6H, t, *J* 7, 2 × CH_3); ^{13}C NMR (100 MHz, $CDCl_3$) δ 62.9 (2 × OCH_2, d, $J_{C\text{-}P}$ 7), 40.8 (CN, d, $J_{C\text{-}P}$ 7), 38.6 (CH_2N, d, $J_{C\text{-}P}$ 7), 23.1 (2 × CH_3, d, $J_{C\text{-}P}$ 5) and 16.3 (2 × OCH_2CH_3, m, $J_{C\text{-}P}$ 7); ^{31}P NMR (162 MHz, $CDCl_3$) δ 12.4; MS CI *m/z* 208 (M + H^+, 100), 131 (10) and 72 (15); HRMS calcd for $C_8H_{19}NO_3{}^{31}P$, 208.1103, found 208.1102.

Diethyl 2-[(*tert*-butyldimethylsilyloxy)methyl]aziridin-1-ylphosphonate 1h

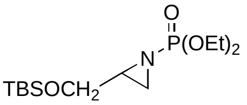

Following **General procedure A** using allyloxy(*tert*-butyl)dimethylsilane (516 mg, 3.0 mmol, Aldrich) gave, following purification of the resulting residue by column chromatography (EtOAc/$CHCl_3$ 1:2), aziridine **1h** as a pale yellow oil (436 mg, 45%). R_f 0.60 (EtOAc/$CHCl_3$ 1:2); ν_{max}/cm^{-1} 2935m, 1470m, 1250s (P=O), 1100m (P–O–C) and 937s; ^{1}H NMR (400 MHz, $CDCl_3$) δ 4.20–4.12 (4H, m, 2 × OCH_2), 3.66–3.64 (2H, m, OCH_2), 2.72–2.64 (1H, m, NCH), 2.34 (1H, dd, *J* 11, 6, CH(*H*)N), 1.99 (1H, dd, *J* 11, 3, C*H*(H)N), 1.59–1.46 (6H, m, 3 × CH_2), 1.33 (6H, t, *J* 7, CH_3), 0.89 (9H, s, $C(CH_3)_3$) and 0.06 (6H, s, $Si(CH_3)_3$); ^{13}C NMR (100 MHz, $CDCl_3$) δ 64.5 ($OCH_{2,}$d, $J_{C\text{-}P}$ 5), 63.4 (2 × $POCH_2$, t, $J_{C\text{-}P}$ 7), 37.3 (CHN, d, $J_{C\text{-}P}$ 6), 28.4 (NCH_2, d, $J_{C\text{-}P}$ 7), 25.9 ($C(CH_3)_3$), 18.4 (SiC), 16.3 (CH_2CH_3, d, $J_{C\text{-}P}$ 6) and -5.3 ($Si(CH_3)_2$); ^{31}P NMR (162 MHz, $CDCl_3$) δ

14.5; MS CI *m/z* 324 (M + H^+, 100), 309 (70) and 97 (25); HRMS calcd for $C_{13}H_{31}NO_4Si^{31}P$, 324.1760, found 324.1762.

Diethyl aziridin-1-ylphosphonate 1i

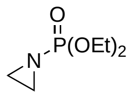

Following the lit. procedure [7], from diethyl chlorophosphate (1.73 g, 10 mmol), 2-chloroethylamine hydrochloride (1.16 g, 10 mmol) and Et_3N (2.8 mL, 20 mmol) in CH_2Cl_2 (50 mL) was obtained a residue which was purified by column chromatography (EtOAc/$CHCl_3$ 1:2) to give aziridine **1i** [7] as a colourless oil (1.15 g, 64%). R_f 0.60 (EtOAc/$CHCl_3$ 1:2); ν_{max}/cm^{-1} 2930s, 1645w, 1460w, 1264s (P=O), 1037s and 967s; ^{1}H NMR (400 MHz, $CDCl_3$) δ 4.16–4.09 (4H, m, 2 × OCH_2), 2.12 (4H, d, *J* 16, 2 × NCH_2) and 1.31 (6H, t, *J* 7, CH_3); ^{13}C NMR (100 MHz, $CDCl_3$) δ 63.4 (2 × $POCH_2$, d, $J_{C\text{-}P}$ 6), 24.3 (CH_2, d, $J_{C\text{-}P}$ 7) and 16.3 (2 × OCH_2CH_3, t, $J_{C\text{-}P}$ 6); ^{31}P NMR (162 MHz, $CDCl_3$) δ 15.4; MS CI *m/z* 180 (M + H^+, 100); HRMS calcd for $C_6H_{15}NO_3{}^{31}P$, 180.0790, found 180.0799.

Diethyl 7-azabicyclo[4.1.0]heptan-7-ylphosphonate 1j

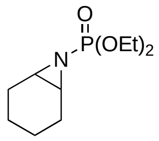

Following **General procedure A** using cyclohexene (246 mg, 3.0 mmol) gave, following purification of the resulting residue by column chromatography (EtOAc/$CHCl_3$ 1:2), aziridine **1j** [5] as a pale yellow oil (133 mg, 19%). R_f 0.55 (EtOAc/$CHCl_3$ 1:2); ν_{max}/cm^{-1} 2936s, 1444m, 1385m, 1232s (P=O), 1020s, 963s and 789; ^{1}H NMR (400 MHz, $CDCl_3$) δ 4.19–4.11 (4H, m, 2 × OCH_2), 2.61 (1H, t, *J* 4, NCH), 2.49 (1H, t, *J* 4, NCH), 1.98–1.77 (4H, m, 2 × CH_2), 1.66–1.60 (2H, m, CH_2), 1.55–1.43 (2H, m, CH_2) and 1.32 (6H, t, *J* 7, 2 × CH_3); ^{13}C NMR (100 MHz, $CDCl_3$) δ 63.3 (2 × OCH_2 d, $J_{C\text{-}P}$ 6), 34.5 (CHN, d, $J_{C\text{-}P}$ 7), 24.2 (2 × CH_2), 20.0 (2 × CH_2) and 16.3 (2 × CH_3 t, $J_{C\text{-}}$

$_P$ 6); ^{31}P NMR (162 MHz, $CDCl_3$) δ 15.3; MS CI *m/z* 234 (M + H^+, 100); HRMS calcd for $C_{10}H_{21}NO_3{}^{31}P$ 234.1259, found 234.1258.

General procedure B: synthesis of N-H aziridinylphosphonates 3

n-BuLi (1.6 M in hexanes, 2.30 mL, 3.7 mmol) was added dropwise to a stirred solution of TMP (0.63 mL, 3.7 mmol) in THF (15 mL) at –78 °C under argon. Following warming to room temperature for 30 min, the resulting solution was re-cooled to –78 °C and a solution of aziridine **1** (0.74 mmol) in THF (1 mL) was added dropwise over 1 min. Following stirring for 1–4 h at –78 °C, saturated aqueous NH_4Cl (2 mL) was added and the flask was warmed to room temperature. The aqueous phase was washed with Et_2O (3 × 10 mL), the combined organic phase was dried ($MgSO_4$) and then evaporated under reduced pressure. Purification of the residue by column chromatography (EtOAc/$CHCl_3$/MeOH 1:1:0.05, SiO_2) gave the aziridinylphosphonate **3**.

Diethyl (2*R,3*R**)-3-butylaziridin-2-ylphosphonate 3a**

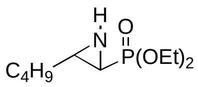

Following **General procedure B** using aziridine **1a** (175 mg, 0.74 mmol) for 4 h gave, following purification of the resulting residue by column chromatography (EtOAc/$CHCl_3$/MeOH 1:1:0.05), aziridinylphosphonate **3a** as a pale yellow oil (159 mg, 91%). R_f 0.40 (EtOAc/$CHCl_3$/MeOH 1:1:0.05); ν_{max}/cm^{-1} 3250br.m (N–H), 2932s, 1653w, 1458m, 1393w, 1233s (P=O), 1127s (P–O–C) and 968s; ^{1}H NMR (400 MHz, $CDCl_3$) δ 4.16–4.07 (4H, m, 2 × OCH_2), 2.33–2.31 (1H, m, CHN), 1.59 (1H, dd, $^2J_{H\text{-}P}$ 18, $^3J_{H\text{-}H}$ 3, CHP), 1.51–1.32 (13H, m, 3 × CH_2, NH, 2 × OCH_2CH_3) and 0.89 (3H, t, *J* 7, CH_3); ^{13}C NMR (100 MHz, $CDCl_3$) δ 62.3 (2 × OCH_2, t, $J_{C\text{-}P}$ 7), 34.9 (CHN), 32.8 (CH_2CH), 29.5 (PCHN, d, $J_{C\text{-}P}$ 139), 29.3 (CH_2), 22.3 (CH_2), 16.4 (2 × CH_3, d, $J_{C\text{-}P}$ 3) and 13.9 (CH_3); ^{31}P NMR (162 MHz, $CDCl_3$) δ 27.33; MS CI *m/z* 236 (M + H^+, 100), 206 (83), 98 (99) and 84 (24); HRMS calcd for $C_{10}H_{23}NO_3{}^{31}P$, 236.1416, found 236.1408.

tert-Butyl (2_R_*,3_R_*)-2-butyl-3-(diethoxyphosphoryl) aziridine-1-carboxylate 14

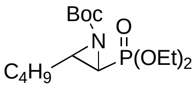

Di-*tert*-butyl dicarbonate (186 mg, 0.85 mmol) was added to a stirred solution of aziridinylphosphonate **3a** (50 mg, 0.21 mmol) and DMAP (29 mg, 0.23 mmol) in CH_2Cl_2 (5 mL) at 0 °C under argon. Following stirring for 2 h, the solution was warmed to room temperature and stirred for 48 h. Saturated aqueous NH_4Cl (2 mL) was added and the organic phase was washed with H_2O (2 mL), dried (Na_2SO_4) and evaporated under reduced pressure. Purification by column chromatography (EtOAc/$CHCl_3$ 1:2) gave *N*-Boc aziridine **14** as a pale yellow oil (61 mg, 85%). R_f 0.64 (EtOAc/$CHCl_3$ 1:2); ν_{max}/cm^{-1} 2980s, 1725s (C=O), 1394m, 1321s, 1257s, 1160s (P=O), 1027s and 970s; ^{1}H NMR (400 MHz, $CDCl_3$) δ 4.24–4.13 (4H, m, 2 × OCH_2), 2.77–2.71 (1H, m, NCH), 2.30 (1H, dd, $^2J_{H\text{-}P}$ 18.5, $^3J_{H\text{-}H}$ 3.5, CHP), 1.58–1.25 (21H, m, $C(CH_3)_3$, 3 × CH_2, 2 × OCH_2CH_3) and 0.92 (3H, t, *J* 7, CH_3); ^{13}C NMR (100 MHz, $CDCl_3$) δ 159.4 (C=O), 81.9 (OC), 62.9 ($POCH_2$, d, $J_{C\text{-}P}$ 6), 62.4 ($POCH_2$, d, $J_{C\text{-}P}$ 6), 41.5 (CN, d, $J_{C\text{-}P}$ 3), 35.3 (CHP, d, $J_{C\text{-}P}$ 197), 30.7 (CH_2CH), 28.9 (CH_2), 27.9 ($C(CH_3)_3$), 22.2 (CH_3CH_2), 16.4 (2 × OCH_2CH_3, t, $J_{C\text{-}P}$ 5) and 13.9 (CH_3); ^{31}P NMR (162 MHz, $CDCl_3$) δ 21.22; MS CI *m/z* 336 (M + H$^+$, 100); HRMS calcd for $C_{15}H_{30}NNaO_5{}^{31}P$ 358.1754, found 358.1756.

Diethyl (2_R_*,3_R_*)-3-(cyclohexyl)aziridin-2-ylphosphonate 3b

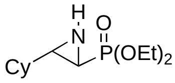

Following **General procedure B** using aziridine **1b** (100 mg, 0.38 mmol, all other reagents were scaled accordingly) for 2 h gave, following purification of the resulting residue by column chromatography (EtOAc/$CHCl_3$/MeOH 1:1:0.05), aziridinylphosphonate **3b** as a pale yellow oil (79 mg, 79%). R_f 0.46 (EtOAc/$CHCl_3$/MeOH 1:1:0.05); ν_{max}/cm^{-1} 3250br.m (N–H), 2927s, 1449m, 1234s (P=O), 1028s and 968s; ^{1}H NMR (400 MHz, $CDCl_3$) δ 4.17–4.09 (4H, m, 2 × OCH_2), 2.16

(1H, m, CHN), 1.88 (1H, d, *J* 12, CHP), 1.75–1.65 (6H, m, 3 × CH_2), 1.35 (6H, t, *J* 7, 2 × CH_3), 1.28–1.07 (5H, m, 2 × CH_2, NH) and 0.98–0.93 (1H, m, C*H*CH_2); ^{13}C NMR (100 MHz, $CDCl_3$) δ 62.5 (OCH_2, d, $J_{C\text{-}P}$ 6), 62.3 (OCH_2, d, $J_{C\text{-}P}$ 6), 41.2 (CHN), 39.9 (CH), 30.9 (CH_2), 30.2 (CH_2), 27.3 (CP, d, $J_{C\text{-}P}$ 201), 26.2 (CH_2), 25.8 (CH_2), 25.6 (CH_2) and 16.5 (2 × CH_3, t, $J_{C\text{-}P}$ 8); ^{31}P NMR (162 MHz, $CDCl_3$) δ 26.87; MS CI *m/z* (rel. int.) 262 (M + H^+, 100), 247 (20) and 124 (20); HRMS calcd for $C_{12}H_{25}NO_3{}^{31}P$, 262.1572, found 262.1561.

Diethyl (2*R,3*R**)-but-3-enylaziridin-2-ylphosphonate 3c**

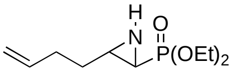

Following **General procedure B** using aziridine **1c** (100 mg, 0.43 mmol, all other reagents were scaled accordingly) for 2 h gave, following purification of the resulting residue by column chromatography (EtOAc/$CHCl_3$/MeOH 1:1:0.05), aziridinylphosphonate **3c** as a pale yellow oil (79 mg, 79%). R_f 0.45 (EtOAc/$CHCl_3$/MeOH 1:1:0.05); ν_{max}/cm^{-1} 3212br.m (N–H), 2931m, 1641s (C=C), 1444m, 1237s (P=O), 1031m and 969s; ^{1}H NMR (400 MHz, $CDCl_3$) δ 5.87–5.79 (1H, m, =CH), 5.01 (2H, dd, *J* 17, 10, CH_2=), 4.16–4.08 (4H, m, 2 × OCH_2), 2.35 (1H, m, NCH), 2.23–2.17 (2H, m, =CHC*H*$_2$), 1.65–1.48 (4H, m, NH, CHP, CH_2) and 1.33 (6H, t, *J* 7, 2 × CH_3); ^{13}C NMR (100 MHz, $CDCl_3$) δ 137.4 (=CH), 115.3 (CH_2=), 62.4 (2 × OCH_2, t, $J_{C\text{-}P}$ 6), 34.4 (CH_2*C*HN), 32.5 (*C*H_2CHN), 31.3 (=CH*C*H_2), 28.7 (CP, d, $J_{C\text{-}P}$ 188), 16.5 (CH_2*C*H_3, d, $J_{C\text{-}P}$ 5) and 16.4 (CH_2*C*H_3, d, $J_{C\text{-}P}$ 6); ^{31}P NMR (162 MHz, $CDCl_3$) δ 26.3; MS CI *m/z* 234 (M + H^+, 100), 219 (35) and 96 (90); HRMS calcd for $C_{10}H_{21}NO_3{}^{31}P$, 234.1259, found 234.1251.

Diethyl (2*R,3*R**)-3-(phenethyl)aziridin-2-ylphosphonate 3d**

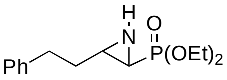

Following **General procedure B** using aziridine **1d** (100 mg, 0.35 mmol, all other reagents were scaled accordingly) for 2 h gave, following purification of the resulting residue by column

chromatography (EtOAc/$CHCl_3$/MeOH 1:1:0.05), aziridinylphosphonate **3d** as a pale yellow oil (87 mg, 87%). R_f 0.42 (EtOAc/$CHCl_3$/MeOH 1:1:0.05); ν_{max}/cm^{-1} 3248br.m (N–H), 2985s, 1604w, 1455m, 1234s (P=O), 1026s, 969s and 700m; ^{1}H NMR (400 MHz, $CDCl_3$) δ 7.29 (3H, m, 3 × C_{Ar}H), 7.21 (2H, m, *2* × C_{Ar}H), 4.17–4.08 (4H, m, 2 × OCH_2), 2.85–2.72 (2H, m, $PhCH_2$), 2.39 (1H, m, NCH), 1.82–1.73 (3H, m, CHP, CH_2), 1.35 (6H, t, *J* 7, 2 × CH_3) and 1.08 (1H, br.s, NH); ^{13}C NMR (100 MHz, $CDCl_3$) δ 141.1 ($C_{Ar\ quat}$), 128.5 (2 × C_{Ar}), 128.4 (2 × C_{Ar}), 126.0 (C_{Ar}), 62.4 (2 × OCH_2, d, $J_{C\text{-}P}$ 6), 34.4 (CHN), 33.7 ($\mathit{C}H_2$CHN), 33.4 ($C_{Ar}\mathit{C}H_2$), 28.5 (CHP, d, $J_{C\text{-}P}$ 136) and 16.5 (2 × CH_3, t, $J_{C\text{-}P}$ 6); ^{31}P NMR (162 MHz, $CDCl_3$) δ 27.26; MS CI *m/z* 284 (M + H^+, 100), 139 (30) and 146 (10); HRMS calcd for $C_{14}H_{23}NO_3{}^{31}P$, 284.1410, found 284.1413.

Diethyl (2*R,3*R**)-3-[4-(*tert*-butyldimethylsilyloxy)butyl]-aziridin-2-ylphosphonate 3e**

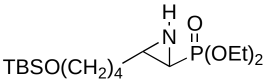

Following **General procedure B** using aziridine **1e** (100 mg, 0.27 mmol, all other reagents were scaled accordingly) for 2 h gave, following purification of the resulting residue by column chromatography (EtOAc/$CHCl_3$/MeOH 1:1:0.05) aziridinylphosphonate **3e** as a pale yellow oil (95 mg, 95%). R_f 0.55 (EtOAc/$CHCl_3$/MeOH 1:1:0.05); ν_{max}/cm^{-1} 3249br.m (N–H), 2932m, 1858s, 1648w, 1473m, 1254m (P=O), 1100s (P–O–C), 1029m and 969s; ^{1}H NMR (400 MHz, $CDCl_3$) δ 4.17–4.10 (4H, m, 2 × OCH_2), 3.61 (2H, t, *J* 6, OCH_2), 2.35 (1H, br.s, CHN), 1.56–1.47 (8H, m, 3 × CH_2, CHP, NH), 1.35 (6H, t, *J* 7, 2 × $OCH_2C\mathit{H}_3$), 0.89 (9H, s, $C(CH_3)_3$) and 0.04 (6H, s, $Si(CH_3)_2$); ^{13}C NMR (100 MHz, $CDCl_3$) δ 62.9 (2 × $POCH_2$, t, $J_{C\text{-}P}$ 7), 62.4 (OCH_2), 34.7 (CHN), 32.8 ($NCH\mathit{C}H_2$), 32.4 ($OCH_2\mathit{C}H_2$), 28.5 (NCHP, d, $J_{C\text{-}P}$ 182), 25.9 ($C(\mathit{C}H_3)_3$), 23.6 (CH_2), 18.3 (SiC), 16.4 ($CH_2\mathit{C}H_3$, t, $J_{C\text{-}P}$ 6) and –5.3 ($Si(CH_3)_2$); ^{31}P NMR (162 MHz, $CDCl_3$) δ 26.5; MS CI *m/z* (rel. int.) 366 (M + H^+, 95), 351 (75), 234 (80) and 228 (100); HRMS calcd for $C_{16}H_{37}NO_4{}^{31}PSi$, 366.2230, found 366.2240.

Diethyl (2*R,3*R**)-3-(4-chlorobutyl)aziridin-2-ylphosphonate 3f**

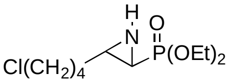

Following **General procedure B** using aziridine **1f** (100 mg, 0.37 mmol, all other reagents were scaled accordingly) for 1 h gave, following purification of the resulting residue by column chromatography (EtOAc/$CHCl_3$/MeOH 1:1:0.05), aziridinylphosphonate **3f** as a pale yellow oil (87 mg, 87%). R_f 0.36 (EtOAc/$CHCl_3$/MeOH 1:1:0.05); ν_{max}/cm^{-1} 3246br.m (N–H), 2985s, 1445m, 1236s (P=O), 1164m (P–O–C), 1028s and 968s; ^{1}H NMR (400 MHz, $CDCl_3$) δ 4.18–4.10 (4H, m, 2 × OCH_2), 3.55 (2H, t, *J* 7, $ClCH_2$), 2.38–2.31 (1H, m, CHN), 1.85–1.80 (2H, m, $ClCH_2CH_2$), 1.67–1.46 (6H, m, 2 × CH_2, NH, CHP) and 1.36 (6H, t, *J* 7, CH_3); ^{13}C NMR (100 MHz, $CDCl_3$) δ 63.5 (2 × OCH_2 t, $J_{C\text{-}P}$ 5), 44.8 ($ClCH_2$), 34.6 (CHN, d, $J_{C\text{-}P}$ 7), 32.3 (CH_2CH), 32.1 ($ClCH_2CH_2$), 28.6 (CHP, d, $J_{C\text{-}P}$ 189), 24.6 (CH_2) and 16.4 (2 × CH_3, t, $J_{C\text{-}P}$ 5); ^{31}P NMR (162 MHz, $CDCl_3$) δ 25.88; MS CI *m/z* 272 (^{35}ClM + H$^+$, 20) and 270 (^{35}ClM + H$^+$, 100); HRMS calcd for $C_{10}H_{22}NO_3{}^{35}Cl^{31}P$, 270.1026, found 270.1013.

Diethyl 3,3-dimethylaziridin-2-ylphosphonate 3g

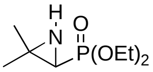

Following **General procedure B** using aziridine **1g** (100 mg, 0.48 mmol, all other reagents were scaled accordingly) for 2 h gave, following purification of the resulting residue by column chromatography (EtOAc/$CHCl_3$/MeOH 1:1:0.05), aziridinylphosphonate **3g** as a pale yellow oil (58 mg, 58%). R_f 0.55 (EtOAc/$CHCl_3$/MeOH 1:1:0.05); ν_{max}/cm^{-1} 3271br.m (N–H), 2985m, 1650w, 1479m, 1234s (P=O), 1129m (P–O–C), 1025m and 798m; ^{1}H NMR (400 MHz, $CDCl_3$) δ 4.15–4.10 (4H, m, 2 × OCH_2), 1.70 (1H, d, *J* 15, CHP), 1.49 (3H, s, CH_3), 1.32 (6H, t, *J* 7, 2 × CH_3) and 1.26 (3H, s, CH_3); ^{13}C NMR (100 MHz, $CDCl_3$) δ 62.2 ($POCH_2$, d, $J_{C\text{-}P}$ 6), 62.0 ($POCH_2$, d, $J_{C\text{-}P}$ 6), 37.6 (CN), 35.8 (CP, d, $J_{C\text{-}P}$ 191) , 26.8 (CH_3), 20.6 (CH_3) and 16.3 (2 × OCH_2CH_3); ^{31}P NMR (162

MHz, $CDCl_3$) δ 25.4; MS CI *m/z* 208 (M + H^+, 90), 100 (45), 70 (100) ; HRMS calcd for $C_8H_{19}NO_3{}^{31}P$, 208.1103, found 208.1099.

(*R*)-1-Aminobutan-2-ol 16

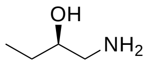

Aq NH_4OH (6.0 mL, 25% w/v) was added to a stirred solution of (*R*)-1,2-epoxybutane (**15**) (0.86 mL, 10 mmol, Aldrich) in MeCN (2 mL) at room temperature. The colourless solution was heated in a sealed tube at 100 °C for 1 h [9]. Following cooling and evaporation, bulb-to-bulb distillation (9 mbar, 125 °C) gave the β-amino alcohol **16** as a colourless oil (622 mg, 70%). $[\alpha]^{20}_D$ = +7.3 (*c* 1.0, $CHCl_3$), lit. [10] $[\alpha]^{25}_D$ = +23.82 (*c* 7.63, MeOH); ν_{max}/cm^{-1} 3353br, m (N–H, O–H), 2928s, 2858m, 1577m, 1467m and 1074w; ^1H NMR (400 MHz, $CDCl_3$) δ 3.46–3.40 (1H, m, OCH), 2.80 (1H, dt, *J* 13, 4, 3, CH(*H*)N), 2.51 (1H, dt, *J* 10, 5, 4 C*H*(H)N), 2.24 (3H, br, NH_2, OH), 1.47–1.39 (2H, m, CH_2CH_3) and 0.94 (3H, t, *J 8*, CH_3); ^{13}C NMR (100 MHz, $CDCl_3$) δ 73.4 (CHO), 46.9 (CH_2N), 27.6 (CH_2CH_3) and 10.0 (CH_3); MS FI *m/z* 90 (M + H^+, 100) and 60 (40); HRMS calcd for $C_4H_{12}NO$, 90.0919, found 90.0917.

Diethyl (*S*)-2-ethylaziridin-1-ylphosphonate 1k

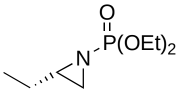

Diethyl chlorophosphate (1.95 mL, 13.5 mmol) was added to a stirred solution of the above prepared β-amino alcohol **16** (600 mg, 6.7 mmol) and Et_3N (2.81 mL, 20.2 mmol) in THF (60 mL) at room temperature under argon. Following stirring for 20 h, NaH (60% w/w dispersion in mineral oil, 1.62 g, 40.4 mmol) was added and the suspension was stirred for a further 16 h. H_2O (0.75 mL) was added and the suspension was filtered through a plug of $MgSO_4$ and washed with Et_2O (100 mL). Following removal of the solvent under reduced pressure, and column chromatography (EtOAc/$CHCl_3$ 1:2), aziridine **1k** was obtained as a pale yellow oil (725 mg, 52%). $[\alpha]^{20}_D$ = +2.32

(c 1.0, $CHCl_3$); R_f 0.55 (EtOAc/$CHCl_3$ 1:2); ν_{max}/cm^{-1} 2982s, 1466m, 1394m, 1260s (P=O), 1032s and 971s; ^1H NMR (400 MHz, $CDCl_3$) δ 4.16–4.09 (4H, m, 2 × OCH_2), 2.48–2.38 (1H, m, NCH), 2.29 (1H, dd, J 10, 6, CH(H)N), 1.88 (1H, dd, J 10, 4, CH(H)N), 1.57–1.40 (2H, m, CH_2), 1.31 (6H, t, J 7, 2 × OCH_2CH_3) and 0.98 (3H, t, J 8, CH_3); ^{13}C NMR (100 MHz, $CDCl_3$) δ 63.2 (2 × OCH_2, t, $J_{C\text{-}P}$ 7), 38.1 (CHN, d, $J_{C\text{-}P}$ 6), 30.5 (CH_2N, d, $J_{C\text{-}P}$ 7), 25.5 (CH_2, d, $J_{C\text{-}P}$ 5), 16.3 (2 × OCH_2CH_3, d, $J_{C\text{-}P}$ 6) and 10.9 (CH_3); ^{31}P NMR (162 MHz, $CDCl_3$) δ 15.3; MS CI m/z 208 (M + H^+, 97), 154 (10), 100 (30), 72 (100) and 96 (90); HRMS calcd for $C_8H_{19}NO_3{}^{31}P$, 208.1103, found 208.1101.

Diethyl (2*S*,3*S*)-3-ethylaziridin-2-ylphosphonate (–)-3k

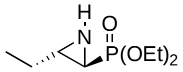

Following **General procedure B** using aziridine **1k** (100 mg, 0.48 mmol, all other reagents were scaled accordingly) for 4 h gave, following purification of the resulting residue by column chromatography (EtOAc/$CHCl_3$/MeOH 1:1:0.05), aziridinylphosphonate (–)-**3k** as a pale yellow oil (89 mg, 89%). The enantiomeric excess (>99% ee) was determined by chiral HPLC analysis of the benzoate derivative, see below. $[\alpha]^{20}_D = -16.5$ (c 1.0, $CHCl_3$); R_f 0.50 (EtOAc/$CHCl_3$/MeOH 1:1:0.05); ν_{max}/cm^{-1} 3289br (N–H), 2985s, 1648m, 1464m, 1394m, 1227s (P=O) and 1026s; ^1H NMR (400 MHz, $CDCl_3$) δ 4.17–4.09 (4H, m, 2 × OCH_2), 2.32 (1H, br.s, CHN), 1.61–1.42 (4H, m, CHP, NH, CH_2), 1.34 (6H, t, J 7, 2 × OCH_2CH_3) and 1.01 (3H, t, J 8, CH_3); ^{13}C NMR (100 MHz, $CDCl_3$) δ 62.4 ($POCH_2$, d, $J_{C\text{-}P}$ 6), 62.3 ($POCH_2$, d, $J_{C\text{-}P}$ 6), 36.1 (CHN), 28.2 (PC, d, $J_{C\text{-}P}$ 186), 26.0 (CH_2), 16.4 (OCH_2CH_3, t, $J_{C\text{-}P}$ 5) and 11.1 (CH_3); ^{31}P NMR (162 MHz, $CDCl_3$) δ 26.5; MS CI m/z 208 (M + H^+, 80), 98 (30), 72 (40) and 70 (100); HRMS calcd for $C_8H_{19}NO_3{}^{31}P$, 208.1103, found 208.1103.

Diethyl (2*S*,3*S*)-1-benzoyl-3-ethylaziridin-2-ylphosphonate (+)-17

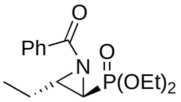

Benzoyl chloride (49 mg, 0.35 mmol) was added to a stirred solution of aziridinylphosphonate (–)-**3k** (60 mg, 0.29 mmol) and Et_3N (49 μL, 0.35 mmol) in CH_2Cl_2 (3 mL) at room temperature under argon. Following stirring for 2 h, H_2O (10 mL) was added and the aqueous phase was washed with CH_2Cl_2 (3 × 5 mL), the combined organic phase was dried ($MgSO_4$) and evaporated under reduced pressure. Purification of the residue by column chromatography (EtOAc/$CHCl_3$ 1:2) gave diethyl (2*S*,3*S*)-1-benzoyl-3-ethylaziridin-2-ylphosphonate (+)-**17** as a colourless oil (81 mg, 90%). $[\alpha]^{20}_D$ = +14.8 (c 1.0, $CHCl_3$); R_f 0.60 (EtOAc/$CHCl_3$ 1:2); ν_{max}/cm^{-1} 3289br.m (N–H), 2985s, 1678s (C=O), 1451w, 1254s (P=O), 1025s, 968m and 723m; ^1H NMR (400 MHz, $CDCl_3$) δ 8.05–8.03 (2H, m, 2 × $C_{Ar}H$), 7.58–7.54 (1H, m, $C_{Ar}H$), 7.47–7.43 (2H, m, 2 × $C_{Ar}H$), 4.20–4.09 (4H, m, 2 × OCH_2), 3.16–3.10 (1H, m, NCH), 2.66 (1H, dd, $^2J_{H-P}$ 18, $^3J_{H-H}$ 3.5, CHP), 1.77–1.66 (1H, m, $CH_3C\mathit{H}(H)$), 1.35 (3H, t, *J* 7, OCH_2CH_3), 1.31 (3H, t, *J* 7, OCH_2CH_3), 1.14–1.03 (1H, m, $CH_3C\mathit{H}(H)$) and 0.97 (3H, t, *J* 8, CH_3); ^{13}C NMR (100 MHz, $CDCl_3$) δ 176.3 (C=O), 133.2 (CAr quat), 132.9 (2 × CAr), 129.1 (2 × CAr), 128.4 (CAr), 63.1 (OCH_2, d, J_{C-P} 6), 62.5 (OCH_2, d, J_{C-P} 6), 44.9 (CHN, d, J_{C-P} 4), 34.4 (PC, J_{C-P} 201), 24.3 (CH_2), 16.4 (OCH_2CH_3, t, J_{C-P} 6) and 11.0 (CH_3); ^{31}P NMR (162 MHz, $CDCl_3$) δ 20.1; MS CI m/z 312 (M + H^+, 15), 174 (100), 156 (40) and 105 (35); HRMS calcd for $C_{15}H_{23}NO_4{}^{31}P$, 312.1365, found 312.1370.

The enantiomeric excess (>99%) was determined by chiral HPLC analysis, Chiralcel OJ-H column (250 × 4.6 mm), 98:2 hexane:*i*-PrOH, 0.25 ml/min. t_R = 76.2 min. Chiral HPLC analysis of racemic diethyl (2*R**,3*R**)-1-benzoyl-3-ethylaziridin-2-ylphosphonate **17**: Chiralcel OJ-H column, 98:2 hexane:*i*-PrOH, 0.25 ml/min. t_R = 71.8 and 90.1 min.

Diethyl (*S*)-(2-aminobutyl)phosphonate (+)-18

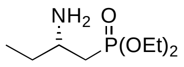

Ammonium formate (304 mg, 4.83 mmol) was added to a stirred suspension of aziridinylphosphonate (–)-**3k** (50 mg, 0.24 mmol) and 10% Pd/C (10 mg, 0.01 mmol) in MeOH (5 mL) at room temperature under argon. Following stirring at reflux for 16 h, the suspension was cooled and NH_4OH (25% aqueous solution) was added until pH > 8. The aqueous phase was washed with CH_2Cl_2 (3 × 5 mL), the combined organic phase was dried ($MgSO_4$) and evaporated under reduced pressure. Purification by column chromatography (CH_2Cl_2 to CH_2Cl_2/MeOH/NH_4OH 96:3:1) gave β-amino phosphonate (+)-**18** as a colourless oil (34 mg, 68%). The enantiomeric excess (>99% ee) was determined by chiral HPLC analysis of the benzoate derivative, see below. $[\alpha]^{20}_D$ = +16.3 (*c* 0.58, MeOH), lit. [11] $[\alpha]^{20}_D$ = +7.3 (*c* 2.3, MeOH, 64% ee), lit. (ent.) [12] $[\alpha]^{20}_D$ = –3.9 (*c* 0.71, CH_2Cl_2, 24% ee); R_f 0.12 (CH_2Cl_2/MeOH/NH_4OH 96:3:1); IR (neat) 3423br.m (N–H), 1643m, 1209w (P=O) and 1025m cm^{-1}; ^1H NMR (250 MHz, CD_3OD) δ 4.20–4.09 (4H, m, 2 × OCH_2), 3.14–3.04 (1H, m, CHN), 2.09–1.99 (1H, m, CH(*H*)P), 1.88–1.80 (1H, m, C*H*(H)P), 1.63–1.45 (2H, m, CH_2), 1.35 (6H, t, *J* 7, 2 × OCH_2CH_3), 0.97 (3H, t, *J* 8, CH_3); ^{13}C NMR (100 MHz, CD_3OD) δ 63.6 ($POCH_2$, d, $J_{C\text{-}P}$ 6), 48.7 (CHN, d, $J_{C\text{-}P}$ 4), 33.6 (PCH_2, d, $J_{C\text{-}P}$ 138), 31.7 (CH_2, d, $J_{C\text{-}P}$ 14), 16.9 (2 × OCH_2CH_3, d, $J_{C\text{-}P}$ 6), 10.5 (CH_3); ^{31}P NMR (162 MHz, CD_3OD) δ 31.4; MS CI *m/z* 210 ($M + H^+$, 100), 193 (30), 153 (35); HRMS calcd for $C_8H_{21}NO_3{}^{31}P$, 210.1259, found 210.1256.

Diethyl (*S*)-2-benzamidobutylphosphonate (–)-19

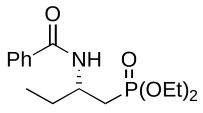

Benzoyl chloride (32 mg, 0.23 mmol) was added to a stirred solution of β-amino phosphonate (+)-**18** (40 mg, 0.19 mmol) and Et_3N (32 μL, 0.23 mmol) in CH_2Cl_2 (3 mL) at room temperature under argon. Following stirring for 2 h, H_2O (10 mL) was added, the aqueous phase was washed with CH_2Cl_2 (3 × 5 mL), the combined organic phase was dried ($MgSO_4$) and evaporated under reduced pressure. Purification by column chromatography (EtOAc/$CHCl_3$ 1:2) gave diethyl (*S*)-2-benzamidobutylphosphonate (–)-**19** as a colourless oil (54 mg, 91%). $[\alpha]^{20}_{D}$ = –28.6 (*c* 0.3, $CHCl_3$); R_f 0.36 (EtOAc/$CHCl_3$ 1:2); IR (neat) 3304br.m (N–H), 2934s, 1713s (C=O), 1579s, 1226s (P=O), 1053s, 959s and 712m cm^{-1}; ^{1}H NMR (400 MHz, $CDCl_3$) δ 7.87–7.84 (2H, m, 2 × $C_{Ar}H$), 7.49–7.38 (3H, m, 3 × $C_{Ar}H$), 4.48–4.32 (1H, m, NCH), 4.20–4.03 (4H, m, 2 × OCH_2), 2.17–2.11 (2H, m, PCH_2), 1.89–1.70 (2H, m, CH_2), 1.34 (3H, t, *J* 7, OCH_2CH_3), 1.28 (3H, t, *J* 7, OCH_2CH_3), 0.98 (3H, t, *J* 8, CH_3); ^{13}C NMR (100 MHz, $CDCl_3$) δ 166.7 (C=O), 134.5 ($C_{Ar\ quat}$), 131.3 (2 × C_{Ar}), 128.5 (2 × C_{Ar}), 127.0 (C_{Ar}), 62.1 (OCH_2, $J_{C\text{-}P}$ 6), 61.5 (OCH_2, $J_{C\text{-}P}$ 6), 46.9 (CHN, $J_{C\text{-}P}$ 6), 29.2 (CHP, $J_{C\text{-}P}$ 139), 28.3 (CH_2, $J_{C\text{-}P}$ 5), 16.4 (2 × OCH_2CH_3, t, $J_{C\text{-}P}$ 6), 10.7 (CH_3); ^{31}P NMR (162 MHz, $CDCl_3$) δ 30.3; MS CI *m/z* 314 (M + H^+, 100), 193 (20), 131 (20), 122 (25), 103 (15), 87 (10); HRMS calcd for $C_{15}H_{25}NO_4{}^{31}P$, 314.1521, found 314.1529.

The enantiomeric excess (>99%) was determined by chiral HPLC analysis, Chiralcel OJ-H column, 98:2 hexane:*i*-PrOH, 0.25 ml/min. t_R = 156.2 min. Chiral HPLC analysis of racemic diethyl 2-benzamidobutylphosphonate **19**: Chiralcel OD-H column, 98:2 hexane:*i*-PrOH, 0.25 ml/min. t_R = 155.4 and 181.7 min.

References

1. Pangborn, A. B.; Giardello, M. A.; Grubbs, R. H.; Rosen, R. K.; Timmers, F. J. *Organometallics* **1996,** *15,* 1518–1520. doi:10.1021/om9503712
2. Gottlieb, H. E.; Kotlyar, V.; Nudelman, A. *J. Org. Chem.* **1997,** *62,* 7512–7515. doi:10.1021/jo971176v
3. Zwierzak, A.; Zawadzki, S. *Synthesis* **1972,** 416–417. doi:10.1055/s-1972-21890
4. Zawadzki, S.; Zwierzak, A. *Tetrahedron* **1973,** *29,* 315–320. doi:10.1016/S0040-4020(01)93296-X
5. Osowska-Pacewicka, K.; Zwierzak, A. *J. Prakt. Chem.* **1986,** *328,* 441–444. doi:10.1002/prac.19863280321
6. Brosius, A. D.; Overman, L. E.; Schwink, L. *J. Am. Chem. Soc.* **1999,** *121,* 700–709. doi:10.1021/ja983013+
7. Osowska-Pacewicka, K.; Zwierzak, A. *Synthesis* **1996,** 333–335. doi:10.1055/s-1996-4213
8. Gajda, T.; Napieraj, A.; Osowska-Pacewicka, K.; Zawadzki, S.; Zwierzak, A. *Tetrahedron* **1997,** *53,* 4935–4946. doi:10.1016/S0040-4020(97)00188-9
9. Hodgson, D. M.; Humphreys, P. G.; Miles, S. M.; Brierley, C. A. J.; Ward, J. G. *J. Org. Chem.* **2007,** *72,* 10009–10021. doi:10.1021/jo701901t
10. Wenghoefer, H. M.; Lee, K. T.; Srivastava, R. M.; Srimannarayana, G.; Clapp, L. B. *J. Heterocycl. Chem.* **1970,** *7,* 1407–1411. doi:10.1002/jhet.5570070631
11. Yuan, C.; Xu, C.; Zhang, Y. *Tetrahedron* **2003,** *59,* 6095–6102. doi:10.1016/S0040-4020(03)00995-5
12. Palacios, F.; Aparicio, D.; Ochoa de Retana, A. M.; de los Santos, J. M.; Gil, J. I.; López de Munain, R. *Tetrahedron: Asymmetry* **2003,** *14,* 689–700. doi:10.1016/S0957-4166(03)00089-2
